# Factors associated with obesity in Iranian elderly people: results from the national health survey

**DOI:** 10.1186/1756-0500-4-538

**Published:** 2011-12-14

**Authors:** Enayatollah Bakhshi, Behjat Seifi, Akbar Biglarian, Kazem Mohammad

**Affiliations:** 1 Department of Statistics and Computer, University of Social Welfare and Rehabilitation Sciences, Tehran, Iran; 2 Department of Physiology, Medicine School, Tehran University of Medical Sciences, Iran; 3 Department of Biostatistics, School of Public Health and Institute of Public Health Research, Tehran University of Medical Sciences, Iran

## Abstract

**Background:**

No studies have been carried out so far to cover the association between factors related to obesity, in a representative sample of the Iranian elderly population.

**Methods:**

The data in this investigation were taken from the National Health Survey in Iran, which included 4380 people aged 60 and older. The logistic regression was applied to model the relationship between the odds of obesity and age, sex, education level, place of residence, smoking and marital status.

**Results:**

Obesity odds ratios were 1.96 (95% CI: 1.53--2.52) for women, 2.16 (95% CI: 1.71--2.72) for the subjects living in urban areas and 0.68 (95% CI: 0.47--0.97) for smokers. Regarding the people aged 60-64 years as the reference group, the obesity odds ratios were 0.86 (95% CI: 0.66--1.10) for ages 65-69 years, 0.75 (95% CI: 0.57--0.97) for ages 70-74 years, 0.43 (95% CI: 0.30--0.60) for ages 75-79 years and 0.36 (95% CI: 0.20--0.63) for ages 80+ years. Using the basic education level as the reference group, obesity odds ratios were 1.38 (95% CI: 1.08-1.76) for the moderate level and 0.92 (95% CI: 0.56- 1.52) for the high level group.

**Conclusions:**

As the result of findings, we are optimistic that we would be able to contribute to the aged community of the society, which could be achieved by means of better treatments and reductions in the rate of obesity.

## Background

Obesity has been called the disease of the twenty-first century. Nearly 1.5 billion adults of age 20 and older are considered to be overweight or obese. Of which, nearly 300 million women and more than 200 million men are obese [1]. The prevalence of obesity is also increasing in older populations throughout the world [2]. The prevalence of obesity among elderly people in industrialized countries estimates suggest ranging from 15% to over 30% [3,4].

Obesity has been reported to be a risk factor for cardiovascular diseases, diabetes, hypertension, some cancers and also some other diseases [5-19]. In general, obesity is related to morbidity, mortality as well as poor quality of life [20]. Although some studies showed that the impact of obesity on mortality may have decreased over time [21], obese elderly people are more likely to become disabled than elderly people at a normal weight [22].

Obesity has been linked to a variety of factors. Himes [23] found that older men are less likely than older women to be obese but Kaplan et al. [24] found that older men are more likely to be obese. Although some studies showed that the prevalence of obesity is going up in all age groups, including older people [25], another study suggested that in industrialized countries, people gain weight up to age of 50-60 and then BMI tends to decrease [26]. Flegal and colleagues have also found that the prevalence of obesity increases from 20 to 60 years of age and decreases after the age of 60 [27].

Another predictor for obesity in elderly people is marital status which has been analyzed in some studies but the findings are not consistent [23,24].

The association of obesity in elderly people with smoking has been analyzed in some studies. An inverse association between smoking and obesity was observed in both sexes [28] but not meaningful among men [24].

Himes found that an inverse relationship between education and obesity [23] and Kaplan et al. showed lower education as a factor which was associated with obesity in both sexes[24].

In Iran, it is commonly believed that overweight and obese people are lazy and gluttonous and they lack self-control. A considerable number of obese people do not go out in public due to the fact that public facilities are very poor comfort levels. For example, the seats in the cinemas are too small for the subject population. Obese people are also more likely to lose the benefits of exercise, which may cause gaining further additional weight. They often feel inferior to others due to the fact that many people would not socialize with an obese person. In Some cases, it is believed that an obese person is taking up more space than he or she should and a job is often refused because of their weights. Until recently, no studies of the association between factors related to obesity have been carried out in a representative sample of the Iranian elderly population. Thus, it is time to invest more to address the issue and the factors related to obesity of the elderly people in this country. The aims of the present study were to explore the associations between age, sex, education level, place of residence, smoking, marital status and obesity among Iranian elderly men and women population. This study was carried out in a sample of men and women representative of the Iranian population older adults (age 60 and over) and adults (aged 20-59).

## Methods

### Data set examined

The National Health Survey in Iran (NHSI) is a survey designed to gain comprehensive knowledge and information about health care problems and difficulties throughout the country, 1999-2000. The survey was conducted under the supervision and with the financial support of the Iranian Ministry of Health and Medical Education. The population sample of the survey consisted of one thousandth of the total Iranian population; non-Iranians were excluded. They were randomly chosen by cluster sampling, each comprising of 8 households. The choice of 8 households for the cluster size was based on one-day performance capacity of the data collection group: Four people (2 physicians, 1 interviewer and 1 lab technician). The statistical framework was based on the household lists, which usually are updated annually and are available to get from every Health Department in the provinces [29-32]. All participants gave their written informed consent for participating the study. Our analytical sample was limited to 2201 women and 2179 men of age 60 and over in the NHSI and 11206 women and 10479 men aged 20-59 years. This study has been approved by the Ethic Committee of the University of Social Welfare and Rehabilitation Sciences.

### Model variables

#### a) Response variable

Height and weight of the sample population were measured. BMI (Body Mass Index) was calculated as weight in kilograms divided by the square of the height in meters (kg/m^2^). Subjects were also classified into obese (BMI ≥ 30 kg/m^2^) and non-obese (BMI < 30 kg/m^2^).

#### b) Independent variables

##### i. Place of residence

The subjects were grouped according to their place of residence as living in cities (urban) or villages (rural).

##### ii. Age (yr)

Information about the respondents' age was based on their self-reported birth year. Adults were stratified into four 10-year age groups (20-29, 30-39, 40-49 and 50-59 years) and older adults into five age groups (60-64, 65-69, 70-74, 75-79 and 80+ years).

##### iii. Education

Education was defined as the total number of years of education. The respondents were categorized into three groups: those with low (0-8 years), moderate (9-12 years) or high (more than 12 years) education levels. The three categories used two dummy variables.

##### iv. Smoking status

Smoking status was dichotomized into smokers vs nonsmokers.

##### v. Marital status

Divorced, widowed and those who have not married were coded as 0 (no spouse) vs others who lived with their spouse was coded 1(spouse).

##### vi. Health shocks

The respondents were grouped according to some health issues including diabetes, high blood pressure, asthma, arthritis and nervous diseases.

### Statistical analysis

The distribution of independent variables and BMI groups were tested with *χ*^2^-test. The logistic regression was applied to model the relationship between the odds of a obesity and age, sex, place of residence, smoking, marital status and the education level. All independent variables were entered into a logistic model simultaneously to assess the predictive ability of each variable while controlling for all other variables. All 2-way interaction terms were dropped from the model because they were not statistically significant.

The results are presented as the odds ratios and their 95% confidence intervals (CI). The Hosmer and Lemeshow test was used in this model to evaluate the significance of improved port with introduction of additional variables. All analyses were carried out by using the SPSS software Package, version 15.

## Results

Among the 2201 female and 2179 male older adult subjects, the mean BMI were 25.13 kg/m^2 ^(SD = 4.96) and 23.64 kg/m^2 ^(SD = 3.92) for females and males, respectively. The mean BMI for urban older adults (n = 2545) was 25.28 kg/m^2 ^(SD = 4.54) and for rural older adults (n = 1835) was 23.15 kg/m^2 ^(SD = 4.23).

Overall, 11.2% of respondents were classified as obese. Table 1 displays the prevalence of obesity within categories of the independent variables. There were statistically significant differences between age groups, men and women, urban and rural, smokers and nonsmokers. Participants who had no spouse were more obese. The prevalence of obesity was higher among older adults who had diabetes, high blood pressure, asthma or arthritis diseases.

**Table 1:** The prevalence of obesity within categories of the risk factors in a random sample of adults age ≥ 60 years (n = 4380), National Health Survey in Iran, using *χ*^2^-test.

Variable	Obesity		
		
	No	%	p-value
**Age group(years)**			
60-64	182	14.1	
65-69	128	12.3	
70-74	117	11.4	< 0.001
75-79	49	6.5	
80+	15	5.6	
**Sex**			
Men	150	6.9	
Women	341	15.5	< 0.001
**Place of Residence**			
Urban	371	14.6	
Rural	120	6.5	< 0.001
**Smoking**			
Nonsmoker	452	12.2	
Smoker	39	5.9	< 0.001
**Marrital Status**			
No spouse	156	12.8	
Spouse	335	10.6	= 0.036
**Education level**			
Basic	342	10.7	
Moderate	128	13.2	= 0.079
High	21	9.9	
**Diabetes**			
No	400	10	
Yes	91	23.5	< 0.001
**High Blood Pressure**			
No	260	8.4	
Yes	231	18.2	< 0.001
**Asthma**			
No	453	10.9	
Yes	38	16.9	= 0.006
**Arthritis**			
No	156	7.5	
Yes	335	14.6	< 0.001
**Nervous Disorders**			
No	452	11.2	
Yes	39	11.2	= 0.98

Turning to the logistic regression analysis, Table 2 summarizes the adjusted odds ratios and their 95% CI's. Using 60-64 years as the reference group, the obesity odds ratios for age groups 65-69, 70-74, 75-79 and 80+ years were 0.86 (95% CI: 0.66-1.10), 0.75 (95% CI: 0.57-0.97), 0.43 (95% CI: 0.30-0.60) and 0.36 (95% CI: 0.20-0.63), respectively.

**Table 2:** Adjusted^a ^odds ratios for obesity^b ^among 4380 Iranian older adults aged ≥60 years, National Health Survey in Iran, in the logistic analysis

variable	OR^c^	95% CI^d^
**Age group(years)**		
60-64	1.00	
65-69	0.86	0.66-1.10
70-74	0.75	0.57-0.97
75-79	0.43	0.30-0.60
80+	0.36	0.20-0.63
**Sex**		
Men	1.00	
Women	1.96	1.53-2.52
**Place of Residence**		
Rural	1.00	
Urban	2.16	1.71-2.72
**Smoking**		
Nonsmoker	1.00	
Smoker	0.68	0.47-0.97
**Marrital Status**		
No spouse	1.00	
Spouse	1.17	0.92-1.48
**Education level**		
Basic	1.00	
Moderate	1.38	1.08-1.76
High	0.92	0.56-1.52

^a ^Adjusted for all other variables in the table.

^b ^Refers to participants with a body mass index ≥ 30 kg/m^2^.

^c ^Odds ratio.

^d ^Confidence interval.

In general, Women were more obese. The obesity odds ratio was 1.96 (95% CI: 1.53-2.52) for women.

An association was observed between place of residence and obesity. The obesity odds ratio was 2.16 (95% CI: 1.71-2.72) for urban participants.

We found a statistically significant inverse association between smoking and obesity. For smoker participants, the adjusted odds ratio was 0.68 (95% CI: 0.47-0.97).

An association was observed between marital status and obesity (but non-significant). Obesity odds ratio was 1.17 (95% CI: 0.92-1.48) for older adults who had spouse.

Using basic education as the reference group, obesity odds ratios for the moderate and high groups were 1.38 (95% CI: 1.08-1.76) and 0.92 (95% CI: 0.56- 1.52), respectively.

To investigate the weight gain across age groups among younger adults, another logistic model was applied, for which the data included 11206 women and 10479 men aged 20-59 years. Table 3 summarizes the adjusted odds ratios and their 95% CI's. Using 20-29 years as the reference group, the obesity odds ratios were 2.29 (95% CI: 2.06--2.56) for ages 30-39 years, 3.21 (95% CI: 2.86--3.60) for ages 40-49 years and 3.13 (95% CI: 2.74--3.58) for ages 50-59 years.

**Table 3:** Adjusted^a ^odds ratios for obesity^b ^among 24017 Iranian adults aged 20-59 years, National Health Survey in Iran, in the logistic analysis

variable	OR^c^	95% CI^d^
**Age group(years)**		
20-29	1.00	
30-39	2.29	2.06-2.56
40-49	3.21	2.86-3.60
50-59	3.13	2.74-3.58
**Sex**		
Men	1.00	
Women	2.76	2.49-3.05
**Place of Residence**		
Rural	1.00	
Urban	2.03	1.85-2.24
**Smoking**		
Nonsmoker	1.00	
Smoker	0.64	0.54-0.75
**Marrital Status**		
No spouse	1.00	
Spouse	1.16	1.05-1.29
**Education level**		
Basic	1.00	
Moderate	0.87	0.78-0.96
High	0.54	0.45-0.65

^a ^Adjusted for all other variables in the table.

^b ^Refers to participants with a body mass index ≥ 30 kg/m^2^.

^c ^Odds ratio.

^d ^Confidence interval.

## Discussion

This cross-sectional study provided findings regarding the possible associations of some factors with obesity in older adults after the age of 60. Using 60-64 years as the reference group, the obesity odds ratios for age groups 65-69, 70-74, 75-79 and 80+ years were 0.86, 0.75, 0.43 and 0.36, respectively. Obesity odds ratios were 1.96 for women, 2.16 for people living in urban areas, and 0.68 for smokers. Using basic education as the reference group, Obesity odds ratios for the moderate and high groups were 1.38 and 0.93, respectively. Some of our results are consistent with other studies. Overall, 11.2% of respondents were obese. The prevalence of obesity in older adult population varies enormously among countries. This prevalence in some Asian and African populations is 0% and in some industrial countries is more than 30% [3,4,33].

The findings show that in Iran people gain weight up to age of around 50-60 years old and after that BMI tends to decrease. Our results on the association between age and obesity are basically in line with some studies [3,24,26,27]. To gain a higher view of the relationship between obesity and age in older people, we have to understand the changes in food intake, energy expenditure, appetite and body composition (that occurs along with ageing) is inevitable. With aging, come bone and muscle losses that influence the body composition. After the age of 30, fat mass increases; whereas fat-free mass (FFM) progressively decreases. FFM (primarily skeletal muscle) decreases by up to 40% from age of 20 to 70 [25,34-36]. Maximal FFM is usually reached at age of 20-30 years, and maximal fat mass is usually reached at age of 60-70. After that, both fat measures subsequently decline [25,34,35]. Therefore, both decrease during old age.

In our present report, women were more likely by 96% to be obese. Elderly women tend to have higher prevalence of obesity than elderly men in most of the Latin American countries [37-39]. Regarding gender, our results are basically in line with these studies.

In our study, obesity was strongly associated with place of residence. Compared to rural elderly subjects, urban elderly subjects were twice as likely to be obese. The environment can influence access to healthy food, lifestyle behaviors such as the trend toward 'eating out', lack of sidewalks and accessible recreation areas. In a cohort study, Glass et al. found that neighborhood conditions can change the patterns of obesity [40]. The finding in the current research agrees with the observations by Gutierrez et al. [41] in some countries in Latin America and a population study conducted in Canada on the relationship between obesity and geographical region [24].

The results in our study suggested an inverse association between smoking and obesity. The inverse association seen between smoking and obesity should not be used to counteract the efforts undertaken against this habit, although smokers often report that they smoke to control their weight. Biological mechanisms as well as psychological factors may be involved. Smoking decreases appetite. Mineur et al. [42] showed that nicotine-induced, that reduces appetite, is due to hypothalamic melanocortin system. Gonseth et al. [43] found that some tobacco companies had added some substances into their cigarette in order to reduce smokers' appetite. An increase of energy expenditure while smoking, both in resting and in light physical activity conditions, may relate to lower prevalence of obesity in smokers. Our results are consistent with the findings of some studies [24,28].

Although we did not find a significant association between marriage and obesity, Iranian elderly people who lived with their spouse were more likely to be obese than their unmarried counterparts. Kaplan et al. found that marriage was positively associated with obesity among older adult women but not men [24]. No evidence was found for an association between obesity and marital status in Taiwanese elderly people [44].

Using basic education as the reference group, our results showed that moderate educational level was associated with a higher likelihood for obesity. The inverse relationships between education status and obesity exist in some societies [3,24]. It is not a straightforward matter to compare those results with ours, due to the different study designs, time spans, regions, and methods of analysis. Our results are consistent with some studies in developing countries [44].

Our results are subjected to some limitations. This study is a cross-sectional study, which means that the direction of these associations cannot be conclusively determined and a causal relationship cannot be inferred but this should be confirmed by further longitudinal studies. We were not able to consider all of the risk factors that may be related to obesity. Income, eating behavior and physical activity were not used in our investigation. The consumption of alcohol is prohibited in Iran. Therefore we had no information on alcohol consumption. However, our study had several strengths. It was performed in a nationally representative sample of the Iranian older adults. To our knowledge, this is the first study that had a sample size sufficient to study factors related to obesity in older adults. This study included men and women aged 60 and over and our findings could be generalized to other people. Height and weight were actually measured rather than self-reported. It is well known that self-reports underestimate the prevalence of obesity [45,46].

## Conclusions

As the result of findings, we are optimistic that we would be able to contribute to the aged community of the society, which could be achieved by means of better treatments and reductions in the rate of obesity.

## Competing interests

The author declares that they have no competing interests.

## Authors' contributions

EB and KM originated the idea for this study, did the research proposal, data analysis and prepared the manuscript. AB co-ordinated the research project, while BS helped and edited the final as the physiology consultant. All authors read and approved the final manuscript.
